# Deep intronic *ANK1* variants causing pseudo‐exon inclusion in hereditary spherocytosis: Whole‐genome sequencing and functional assessment

**DOI:** 10.1111/bjh.70703

**Published:** 2026-07-16

**Authors:** Victor Marin, Alexandre Janin, Céline Renoux, Yoann Huguenin, AURAGEN consortium, Stéphanie Dulucq, Louis Lebreton, Philippe Joly, Julian Boutin

**Affiliations:** ^1^ Laboratory of Biochemistry University Hospital Centre Bordeaux Bordeaux France; ^2^ GCS AURAGEN Lyon France; ^3^ Service de Biochimie et de Biologie Moléculaire, Centre de Biologie et de Pathologie Est Hospices Civils de Lyon Bron France; ^4^ Pellegrin Hospital Group Children's Hospital, Pediatry Bordeaux University Hospital Centre Bordeaux Bordeaux France; ^5^ Laboratory of Hematology University Hospital Centre Bordeaux Bordeaux France; ^6^ University of Bordeaux, Inserm, UMR1312, BRIC, BoRdeaux Institute of Oncology Aquitaine France

**Keywords:** ANK1, deep intronic variants, hereditary spherocytosis, pseudo‐exon, whole‐genome sequencing

To the Editor,

Hereditary Spherocytosis (HS) is a congenital haemolytic anaemia characterized by osmotically fragile, spherical red blood cells (RBCs).^1^ It results from defects in RBC membrane–cytoskeleton proteins. Reduced surface‐to‐volume ratio and cellular deformability lead to premature splenic sequestration and destruction, causing anaemia, jaundice and splenomegaly of variable severity. While genetically heterogeneous, HS is most commonly caused by *ANK1* mutations, responsible for approximately 50% of cases^1^, ^2^, ^3^. *ANK1* encodes ankyrin‐1, a multi‐domain structural protein linking the spectrin‐actin cytoskeleton to the transmembrane anion exchanger 1 (Band 3), thereby ensuring the mechanical stability and biconcave morphology of the RBC^4^. Ankyrin‐1 deficiency destabilizes this vertical interaction, leading to HS. The established *ANK1* mutational spectrum predominantly consists of coding region variants or canonical splice‐site mutations^2^, ^5^. Broad access to whole‐genome sequencing (WGS) has revealed deep intronic variants (located >100 bp from splice junctions) as an emerging cause of monogenic disorders often missed by standard panels^6^, ^7^. Their pathogenicity typically arises from the creation of cryptic splice sites, which could result in the aberrant inclusion of an intronic sequence (a ‘pseudo‐exon’ [PE]) into the mature messenger Ribonucleic acid (mRNA). This event frequently disrupts the reading frame, introduces a premature termination codon (PTC) and ultimately leads to a significant reduction in the level of functional protein^8^. While this mechanism was established in the *hema6* murine model,^9^ human deep‐intronic *ANK1* variants causing pseudo‐exon inclusion have never been documented in HS. Here, we describe two cases of HS associated with heterozygous deep intronic *ANK1* variants identified by WGS and functionally assessed by diagnosis splice assays, highlighting the importance of considering non‐coding regions in the genetic diagnosis of unresolved cases of haemolytic anaemia.

The first case was a young man, now 18, who was diagnosed with HS at the age of two after presenting with asthenia. The clinical presentation was consistent with a de novo or recessive form of HS, with no family history. His clinical course was marked by significant haemolytic anaemia (haemoglobin [Hb] 5.7–8.7 g/dL), requiring three transfusions at age four after Parvovirus B19 aplastic crisis and splenectomy at age six for major splenomegaly. Post‐splenectomy, his Hb levels stabilized above 13.0 g/dL, with only biochemical evidence of compensated residual haemolysis (Table S1). Complications included a history of cholelithiasis, which resolved with ursodeoxycholic acid treatment. Initial diagnostic tests, including osmotic gradient ektacytometry, the presence of spherocytes on the blood film and eosin‐5′‐maleimide (EMA) binding, confirmed the HS diagnosis (Table S1). A first‐tier genetic analysis by a short‐read Next‐Generation Sequencing (NGS) panel was inconclusive, identifying only a heterozygous variant of unknown significance (VUS) in the *EPB42* gene (encoding erythrocyte membrane protein band 4.2; NM_001114134.2: c.1115G>A), a finding that did not explain the severe phenotype (Figure 1A).

**FIGURE 1:**
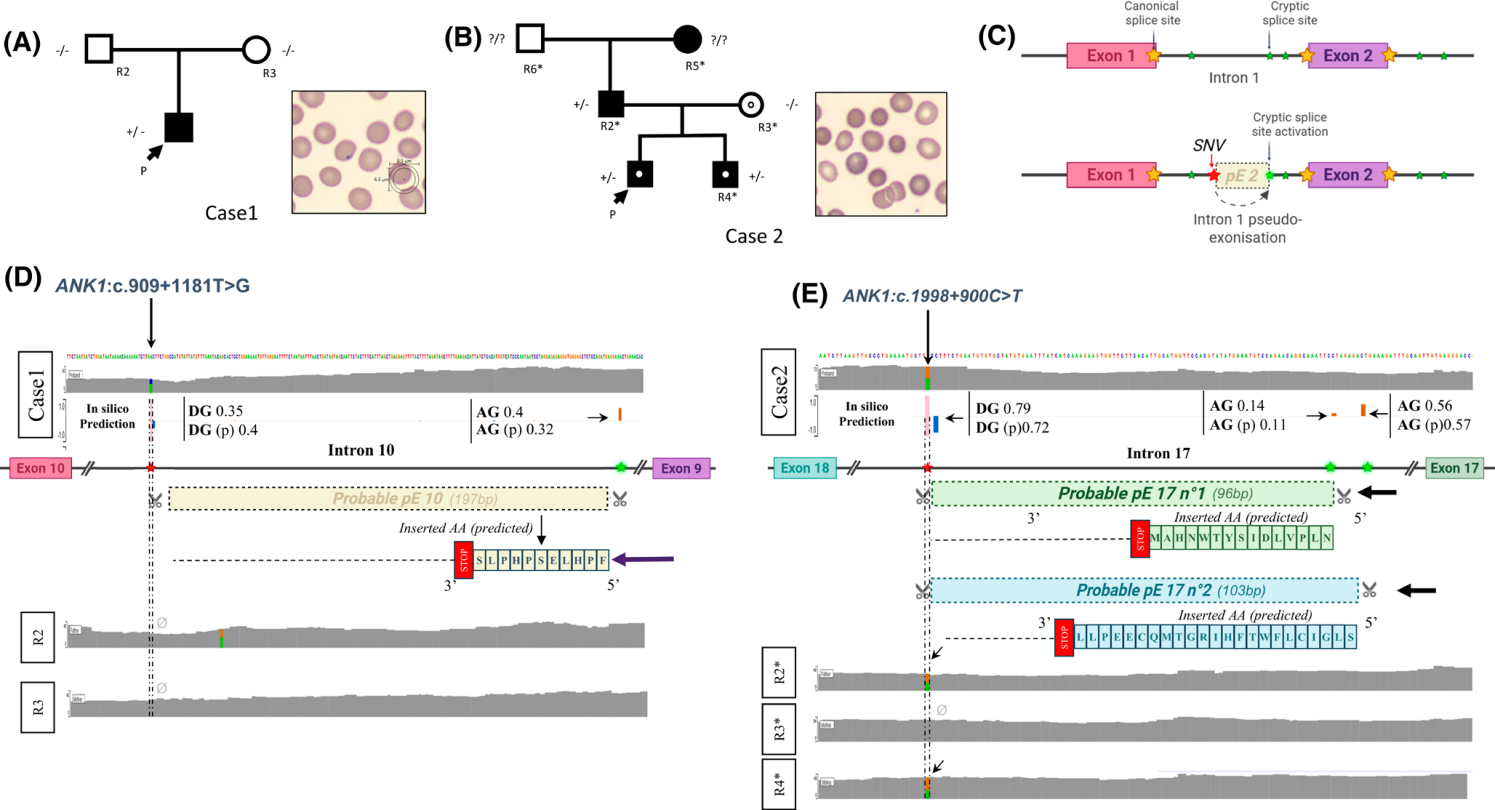
Genomic and familial evidence for pathogenic deep‐intronic *ANK1* variants. Integrated views for two families combining pedigrees, variant location with *in silico* splice predictions and confirmatory whole‐genome sequencing (WGS) alignments. *Squares, males; circles, females; filled symbols, affected; arrow, proband. Pe, pseudo‐exon*. (A) Family pedigree of Case 1 with peripheral blood film (May–Grünwald–Giemsa, MGG) showing spherocytes. Whole‐genome sequencing (WGS) identifies *ANK1* c.909+1181T>G (+) in the proband; segregation with available relatives (R2, R3) supports involvement in the phenotype. (B) Family pedigree of Case 2 with MGG smear. WGS shows *ANK1* c.1998+900C>T (+) transmitted from the carrier father (R2*), present in the affected proband and brother (R4*). The central dot indicates co‐inheritance of a *G6PD* mutation. (C) Model of pseudoexon activation. Top: Canonical *ANK1* pre‐mRNA splicing connects exon 1 to exon 2. Bottom: An intronic single‐nucleotide variant (SNV) strengthens latent (cryptic) 3′/5′ splice sites within intron 1, resulting in recognition of an intronic fragment as a pseudoexon (pE) and its aberrant inclusion between exons 1 and 2. Green stars = cryptic sites, Yellow star = canonical site (D) Case 1: ANK1:c.909+1181T>G (intron 10). Integrative Genomic Viewer (IGV) snapshot showing short‐read alignments across exon 10 and flanking intron 10. The variant position is marked by the arrow. *In silico* splice analysis predicts creation/strengthening of a donor and an acceptor site (DG/AG scores indicated), defining a putative intron‐10 pseudoexon (pE10; beige dashed box). Translation of the inserted sequence is predicted to add novel amino acids followed by a premature stop codon (red ‘STOP’). Coverage tracks from relatives (R2, R3) are shown below for comparison. (E) Case 2: ANK1:c.1998+900C>T (intron 17). IGV snapshot across exon 18 and intron 17 with the variant indicated by the arrow. Splice‐site predictions support activation of cryptic splice sites and two alternative pseudoexons within intron 17 (pE17 #1 and pE17 #2; green/blue dashed boxes). Both predicted insertions would introduce novel amino‐acid sequences and an early termination codon (red ‘STOP’). Coverage from family members (R2*, R3*, R4*) is displayed for reference and show variant segregation.

Similarly, a second patient (Case 2) presented with a comparable diagnostic challenge. This boy was diagnosed 15 days after birth with severe haemolytic anaemia (Hb = 6.0 g/dL), requiring three blood transfusions before the age of 6 months and weekly erythropoietin therapy during the first 9 months of life. A strong paternal family history of autosomal dominant HS was noted, with his father, paternal grandmother and one brother also affected. A maternal family history of glucose‐6‐phosphate dehydrogenase (G6PD) deficiency was present, and the patient was confirmed to have a hemizygous *G6PD* variant (Class B) (Viangchan, NM_001360016.2:c.871G>A). The blood film showed characteristic spherocytes and the EMA binding test confirmed the HS diagnosis. Baseline Hb fluctuated between 8.0 and 8.5 g/dL, and he experienced significant clinical repercussions, including marked asthenia, exercise intolerance and persistent jaundice. He suffered a severe aplastic crisis secondary to a Parvovirus B19 infection with a Hb nadir of 3.2 g/dL requiring two further transfusions at the age of 5. A total splenectomy was performed at the age of six, resulting in overall clinical improvement, with Hb levels normalizing above 12.0 g/dL (Table S1). Initial genetic testing identified two VUS in the *SPTA1* gene (encoding erythrocytic spectrin alpha 1; NM_003126.4:c.1982G>A and c.3357G>C), all maternally inherited (Figure 1B). Given these remaining molecular diagnostic dilemmas, WGS was performed for both patients within the framework of the French genomic medicine programme and written informed consent for genetic analysis and publication of de‐identified clinical and molecular data was obtained (Supporting Information: Methods). In both patients, rare deep intronic single‐nucleotide variants (SNVs) in *ANK1* were identified as strong candidate causes of HS. These variants, located far from the canonical splice sites, would not have been detected by standard exome or panel‐based sequencing and can result in cryptic splice site modulation leading to various functional effects (Figure 1C).

In Case 1, we identified a *de novo* deep intronic *ANK1* variant (NM_000037.4:c.909+1181T>G) absent from GnomAD v4 population database. *In silico* prediction tools suggested a relative probability of aberrant splicing through the creation of a novel acceptor splice site (acceptor gain, AG = 0.40) and a novel donor splice site (donor gain, DG = 0.35) according to SpliceAI^10^ 10 k score, while SPiP^11^ predicted a 30.67% risk of PE creation. The predicted consequence is the inclusion of a 197 bp PE between exons 9 and 10, leading to a PTC 12 codons downstream (Figure 1D).

In Case 2, trio analysis revealed the deep intronic variant *ANK1* NM_000037.4:c.1998+900C>T, which was inherited from the father and segregated with the disease phenotype. This variant is absent from GnomAD v4 population database. *In silico* analysis predicted two possible outcomes. A major one (SpliceAI 10 k score AG = 0.56/DG = 0.79, SPiP 96.7% risk of PE creation), with the creation of a 103 bp pseudo‐exon between exons 17 and 18, leading to a PTC 24 codons downstream or a possible minor one (96 bp) with a PTC 16 codons downstream (AG = 0.14) (Figure 1E). Pangolin^12^ and AlphaGenome^13^ supported the SpliceAI‐prioritized pseudo‐exon model. AlphaGenome modelling further predicted, for the ALT allele, a directional increase in donor/acceptor splice‐site probability and in cryptic‐junction usage. This predicted increase was observed in haematopoietic and erythroid/progenitor‐relevant tracks (e.g. K562) and was consistent across all cellular contexts examined (Figure S1, Supporting Information: Methods).

Functional minigene assays were performed as previously described^14^ (Figure 2A, Supporting Information: Methods). In case 1, the mutated construct showed a near‐complete disappearance of the normally spliced transcript (247 bp) and the appearance of two aberrant transcripts, a major one of 444 bp (197 bp PE insertion) and a minor one of 282 bp (35 bp PE insertion). This second PE was not predicted by SpliceAI and leads to a PTC 5 codons downstream (Figure 2B). This result supports a splice‐disruptive effect of the c.909+1181T>G variant through activation of cryptic splice sites, providing functional evidence for pathogenicity. In case 2, the mutated construct produced a single aberrant transcript of 350 bp, with a complete absence of the normally spliced 247 bp product. This indicates that the c.1998+900C>T variant results in complete loss of the canonical product in the minigene assay, through inclusion of the 103 bp pseudo‐exon predicted by SpliceAI (Figure 2C). A reverse transcription polymerase chain reaction (RT‐PCR) on patient blood Ribonucleic acid (RNA) confirmed the presence of the same aberrant transcript with similar ratios of expression on gels and sashimi‐plots, supporting the in vitro findings and validating the splicing defect in vivo (Figure 2D,E).

**FIGURE 2:**
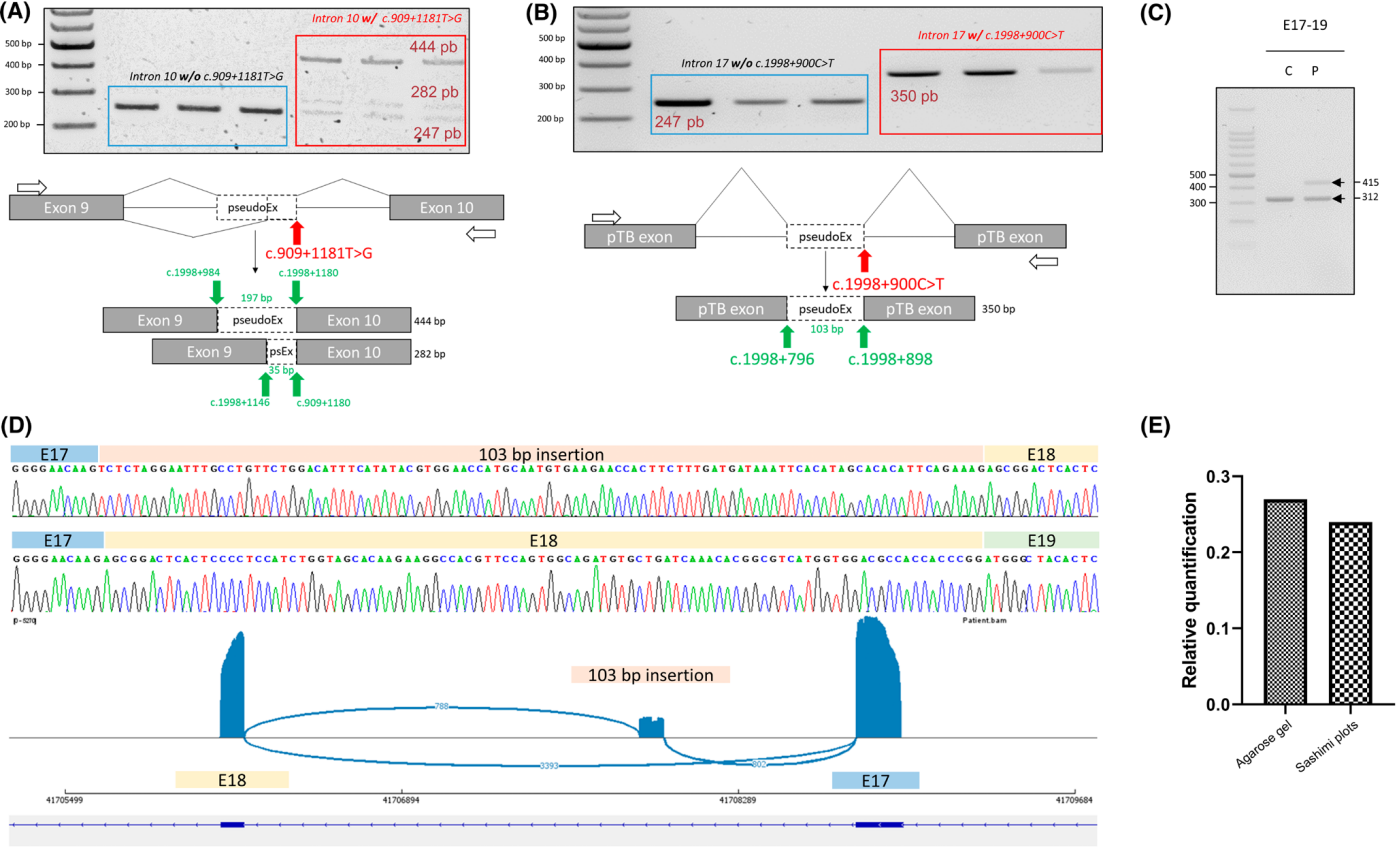
Minigene assay and RNA‐seq confirm pseudo‐exon inclusion and loss of canonical splicing. (A) RT‐PCR products obtained from HeLa cells transfected with wild‐type (WT) or mutant constructs for c.909+1181T>G, three products are observed: 247 bp WT, 282 bp mixed and 444 bp pseudo‐exon‐containing fragment; the mutant lane is dominated by the 444 bp band. (w/ = with; w/o = without) (B) RT‐PCR products obtained from HeLa cells for c.1998+900C>T, only an aberrant 350 bp product is detected. M, 100‐bp ladder. (C–E) ANK1 mRNA study on RNA extracted from peripheral blood leukocytes of the proband carrying c.1998+900C>T variant. (C) Amplification of the region spanning between exons 17 and 19 confirmed an aberrant product (415 bp) containing the 103 bp insertion, as shown by long read sequencing (D). (E) The relative quantification showed a similar ratio between the insertion and the normal transcript for agarose gel quantification or by Sashimi plot analysis.

Both patients harbour rare heterozygous deep intronic variants that generate pseudo‐exon inclusion and ankyrin‐1 haploinsufficiency. The pseudo‐exon insertions are predicted to introduce PTCs, consistent with loss of canonical *ANK1* function. Together with rarity, segregation/*de novo* status, phenotype specificity and functional evidence, these findings support the American College of Medicale Genetics/Association for Molecular Pathology (ACMG/AMP) pathogenic or likely pathogenic classification^15^. Minigene transcript ratios may provide a hypothesis‐generating explanation for disease severity differences: Case 2 showed complete loss of the canonical product in vitro and severe familial HS, whereas Case 1 retained a minor canonical fraction and achieved near‐normal Hb levels after splenectomy.

Given the two‐patient cohort, the non‐erythroid minigene context and the complexity of genotype–phenotype correlations in HS, this interpretation remains exploratory. Splice predictors provide supportive evidence but do not replace patient‐derived transcriptomic validation. Patient‐derived CD34^+^ progenitors differentiated along the erythroid lineage would constitute an alternative relevant validation approach, while patient‐derived whole‐blood RNA, when available, provides complementary in vivo evidence from the haematopoietic compartment.

From a diagnostic standpoint, these cases underscore three critical insights. First, they demonstrate the superiority of genome‐wide analysis over exome‐based strategies since both variants lie >900 bp from the nearest exon and would have been missed by ‘exon‐centred’ targeted panels. Complete intronic targeted panel or WGS coupled with dedicated splice‐prediction pipelines is therefore key for ‘unsolved’ membranopathies. Second, functional validation remains imperative, as minigene assays can rapidly convert a VUS into a fully characterized pathogenic variant, with results that may differ from *in silico* predictors. Third, molecular diagnosis has direct clinical relevance, offering families clarity on recurrence risks and guiding long‐term management, including transfusion planning and the assessment of splenectomy efficacy. These cases represent first examples of human *ANK1* variants located several 100 base pairs from canonical splice junctions and functionally shown to activate pseudo‐exon inclusion in HS. They extend the *ANK1* mutational spectrum and highlight the contribution of cryptic splicing to RBC disorders. We propose that deep‐intronic *ANK1* variants should be systematically considered in the molecular work‐up of unresolved hereditary haemolytic anaemia, particularly when first‐tier short‐read NGS panels or exomes are inconclusive.

## AUTHOR CONTRIBUTIONS

**Alexandre Janin:** Investigation; formal analysis; writing – review and editing; methodology; resources; visualization. **AURAGEN consortium:** Methodology; software; formal analysis; writing – review and editing. **Yoann Huguenin:** Resources; investigation; writing – review and editing. **Céline Renoux:** Methodology; investigation; validation; writing – review and editing. **Victor Marin:** Conceptualization; data curation; investigation; formal analysis; project administration; visualization; writing – original draft; writing – review and editing. **Philippe Joly:** Investigation; methodology; writing – review and editing. **Stéphanie Dulucq:** Methodology; investigation; validation; writing – review and editing. **Louis Lebreton:** Methodology; investigation; validation; writing – review and editing. **Julian Boutin:** Supervision; project administration; writing – original draft; writing – review and editing; conceptualization; investigation; validation; formal analysis.

## CONFLICT OF INTEREST STATEMENT

The authors have no conflicts of interest.

## FUNDING INFORMATION

This research did not receive any specific grant from funding agencies in the public, commercial, or not‐for‐profit sectors.

## Supporting information


Supplementary Material (Methods, References, Table S1, Figure S1)

## Data Availability

The data that support the findings of this study are available on request from the corresponding author. The data are not publicly available due to privacy or ethical restrictions.
