# Supplementary material for: Deep intronic ANK1 variants causing pseudo‐exon inclusion in hereditary spherocytosis: Whole‐genome sequencing and functional assessment
Source: Br J Haematol. 2026 Jul 16;209(4):1950–4. doi: 10.1111/bjh.70703 (PMC13674142; doi:10.1111/bjh.70703)
Supplement: Supplementary file 1 [file BJH-209-1950-s001.docx]

Supplementary Material

**Supplementary Methods**

Next-Generation Sequencing (NGS)

For this study, genomic DNA was extracted from whole blood sample. Two separate approaches were employed. First a targeted panel focusing on 55 genes associated with congenital hemolysis was performed following the protocol previously described ^1,2^. Then Whole genome sequencing was performed following the recommendations of France Genomic Medicine Plan. Whole blood extracted genomic DNA was sequenced according to standard procedures for a PCR-Free genome on a NovaSeq6000 instrument (Illumina). Sequencing data were aligned to the GRCh38p13 full assembly using bwa 0.7+. Variants were called by several algorithms including GATK4+, Bcftools1.10+, Manta1.6+, CNVnator0.4+, and annotated using the variant effect predictor. Detected variants were prioritized using in-house procedures. Following WGS, variants were prioritized within the AURAGEN consortium framework using a multi-step strategy integrating variant rarity, predicted functional impact, gene–disease validity, inheritance model, segregation data when available, and HPO-driven phenotype matching. When no definitive diagnosis was obtained from this first-level analysis, the complete WGS dataset was further reviewed using iterative phenotype- and inheritance-driven filters, including dominant/de novo, autosomal recessive, compound heterozygous, X-linked, CNV/SV and non-coding splice-oriented models. Rare intronic variants in phenotype-relevant genes were retained when supported by segregation, biological plausibility and convergent splice-prediction evidence. Further details are available on request on http://www.auragen.fr

Minigene Assay

The functional consequence of the variant on pre-mRNA splicing was determined using an in vitro minigene assay as previously described ^3^. A genomic fragment of *ANK1* gene of 552 and 688 bp respectively, encompassing variation carrying intron 10 & 17 was cloned into the pTB2 splice-reporter vector in order to obtain a wild-type and a mutant vector for each variation. Forty-eight hours after transfection in HeLa cells, total RNA was extracted. RT‐PCR was performed using random primers. Complementary DNA (cDNA) amplification was performed using vector‐specific primers surrounding the cloning site. The PCR products were resolved on a 2% agarose gel and sequenced to identify splicing events. All transfection experiments were performed in triplicate.

RNA isolation and RT‐PCR assay for c.1998+900C>T variant

Blood was collected in a PAXgene blood RNA tube (PreAnalytix, Qiagen). Total cellular RNA was extracted using the PAXgene blood RNA kit according to the manufacturer’s procedure. The isolated *ANK1* mRNA was reverse-transcribed to cDNA using hexamers and Transcriptor High Fidelity cDNA Synthesis kit (Roche) according to the manufacturer’s recommendations. *ANK1* cDNA was amplified by PCR thanks to a forward primer targeting exon 17 (5’ AATGGCTACACCCCTTTGCA 3’) and a reverse one targeting exon 18 and 19 (5’ GGGAGTGTAGCCCATCCGG 3’) with HotStar Taq DNA polymerase (Qiagen) in the following cycling conditions: a first activation step of 15 min at 95°C, 35 cycles of 20 s at 95°C, 20 s at 55°C, and 1 min at 72°C and the last elongation step of 5 min at 72°C. The RT‐PCR products were separated on 2% agarose gel. After a cloning step of each RT-PCR products thanks to TOPO TA Cloning Kit according to the manufacturer’s procedure, sequencing was performed. The same procedure was performed on a control sample.

ONT Sequencing

Library preparation was performed using Rapid sequencing barcoding kit BC1-96 (Oxford Nanopore Technologies). After a quantification step (Qubit 1X dsDNA HS Assay Kit, Thermo Fischer Scientific), the library was loaded onto MinION Flowcells, according to the manufacturer’s instructions (FLO-MIN114, R10.4.1; Oxford Nanopore Technologies), and performed on MinION device (Mk1B; Oxford Nanopore Technologies).

**Supplementary References**

1.	Richards S, Aziz N, Bale S, Bick D, Das S, Gastier-Foster J, et al. Standards and guidelines for the interpretation of sequence variants: a joint consensus recommendation of the American College of Medical Genetics and Genomics and the Association for Molecular Pathology. Genet Med. 2015;17:405–24. doi:10.1038/gim.2015.30 PubMed PMID: 25741868; PubMed Central PMCID: PMC4544753.

2.	Marin V, Lebreton L, Guibet C, Mesli S, Redonnet-Vernhet I, Dexant M, et al. Case report: Unveiling genetic and phenotypic variability in Nonketotic hyperglycinemia: an atypical early onset case associated with a novel GLRX5 variant. Front Genet. 2024;15:1432272. doi:10.3389/fgene.2024.1432272 PubMed PMID: 39323869; PubMed Central PMCID: PMC11422140.

3.	Janin A, Chanavat V, Rollat-Farnier PA, Bardel C, Nguyen K, Chevalier P, et al. Whole MYBPC3 NGS sequencing as a molecular strategy to improve the efficiency of molecular diagnosis of patients with hypertrophic cardiomyopathy. Hum Mutat. 2020;41:465–75. doi:10.1002/humu.23944 PubMed PMID: 31730716.

**Supplementary Table S1: Clinical and hematologic presentation of the 2 cases**

| Parameter | Case 1 (Sporadic Form) | Case 2 (Familial Form) | Reference values |
| --- | --- | --- | --- |
| Age at Diagnosis | 2 years | 15 days |  |
| Presenting Symptoms | Asthenia | Severe hemolytic anemia |  |
| Splenomegaly | Yes (Major) | Yes (10 cm axis pre-splenectomy) |  |
| Splenectomy | + (Age 6) | + (Age 6) |  |
| HS-Related Complications | Cholelithiasis | None reported |  |
| Other Major Co-morbidities | None reported | Pyloric stenosis, pyelonephritis |  |
| Transfusions | + (3), incl. 2 for aplastic crisis (Parvo B19) | + (5), incl. 2 for aplastic crisis (Parvo B19) |  |
| Specific Therapy | Folates | EPO (from 3 weeks to 9 months) + Folates |  |
| --- Labs (Pre-Splenectomy) --- |  |  |  |
| Hb (g/dL) | 5.7 – 8.7 | 8.0 – 8.5 (nadir at 3.2) | [11.3–15.7] |
| Total Bilirubin (µmol/L) | N/A | 38 | [5–20] |
| --- Labs (Post-Splenectomy) --- |  |  |  |
| Hb (g/dL) | 15.9 | 12.4 | [11.3–15.7] |
| Reticulocytes (G/L) | 54 | 55 | [20–80] |
| LDH (U/L) | 284 | 223 | [125–220] |
| Haptoglobin (g/L) | 0.15 | 0.12 | [0.35–2.50] |
| Total Bilirubin (µmol/L) | 14 | 7 | [5–20] |
| --- Specific Tests --- |  |  |  |
| EMA Binding Test | Positive | Positive |  |
| Osmotic gradient ektacytometry | Positive |  |  |
| Blood Smear | Spherocytes | Spherocytes |  |
| First-Line Genetics | NM_001114134.2:c.1115G>A | NM_003126.4:c.1982G>A and c.3357G>C) |  |
| Co-inheritance? | No | Yes (GPD6 deficiency) |  |

**Figure S1. AlphaGenome splice-site predictions and per-biosample junction fold-change for deep-intronic ANK1 variants.**

Each variant is analysed independently. (A, C) AlphaGenome-predicted SPLICE_SITES probability on the ANK1 minus strand (donor, top; acceptor, bottom) over a ±1 kb window centered on the variant; REF (grey, dashed) and ALT (case color, solid) traces are overlaid. Donor/acceptor labels follow transcript orientation; the x-axis uses plus-strand genomic coordinates (bp offset from the variant). Yellow shading marks the minigene-defined pseudo-exon (197 bp, Case 1; 103 bp, Case 2). (B, D) log₂(ALT/REF) for the top-predicted cryptic junction across 15 biosample-specific RNA-seq tracks (hematopoietic/progenitor-associated, case colour; non-hematopoietic controls, grey). pA, polyA-selected RNA-seq; tot, total RNA-seq.
